# Evidence-based antioxidant activity of the essential oil from Fructus A. zerumbet on cultured human umbilical vein endothelial cells’ injury induced by ox-LDL

**DOI:** 10.1186/1472-6882-12-174

**Published:** 2012-10-07

**Authors:** Xiang-chun Shen, Ling Tao, Wan-kui Li, Yan-yan Zhang, Hong Luo, Yu-yi Xia

**Affiliations:** 1 Research Division of Pharmacology, Guiyang Medical University, Guiyang 55004, People’s Republic of China; 2 Department of Pharmaceutics, Guiyang Medical University, Guiyang 55004, People’s Republic of China

**Keywords:** Essential oil, Fructus Alpiniae zerumbet, Human umbilical vein endothelial cells, Oxidized low-density lipoprotein, Oxidative stress

## Abstract

**Background:**

The essential oil from Fructus Alpiniae zerumbet (FAZ) is its principal bioactive ingredient, and is widely used in *Miao* folk herbs in Guizhou province for the treatment of gastrointestinal and cardiovascular diseases. Several studies have confirmed that FAZ ameliorates hyperlipidemia and atherosclerosis. Because endothelial dysfunction often accompanies cardiovascular diseases, especially hyperlipidemia and atherosclerosis, the present study concentrated on evaluating the endothelial protective effects of the essential oil from FAZ (EOFAZ) on oxidized low-density lipoprotein (ox-LDL)-induced injury of cultured human umbilical vein endothelial cells (HUVECs) and on the regulation of oxidative stress.

**Methods:**

Cell viability was analyzed with the MTT assay and trypan blue exclusion staining (TBES). Cell injury was assessed by lactate dehydrogenase (LDH) release. Biochemical enzymatic methods were used to evaluate the oxidative stress, including the lipid peroxidation product, malondialdehyde (MDA), reduced glutathione (GSH), superoxide dismutase (SOD), catalase (CAT) and glutathione peroxidase (GSH-Px).

**Results:**

The redox status of HUVECs was significantly exacerbated after exposure to ox-LDL. EOFAZ protected HUVECs against ox-LDL injury as assessed by the MTT assay, TBES and LDH release. Furthermore, EOFAZ ameliorated the oxidative stress by elevating the activities of SOD, CAT and GSH-Px, and increasing the GSH levels, in addition to attenuating the MDA contents.

**Conclusions:**

The present data provide the first experimental evidence that EOFAZ protects endothelial cells against ox-LDL-induced injury, and indicate that this protection involves ameliorating the redox status.

## Background

It is well known that atherosclerosis (AS) is the leading cause of death in most Western countries, and is a crucial pathological factor in the development of cardiovascular diseases, leading to alterations and lesions in the inner walls of blood vessels [1]. Endothelial cells are crucial for maintaining the physiological functions of the cardiovascular system, and endothelial dysfunction has been implicated in the initiation and propagation of AS processes [2]. Increasing evidence suggests that stress by an imbalanced cellular activity of production and elimination of reactive oxygen species (ROS) is involved in the pathophysiology of AS [3]. Although its etiology is multifactorial, endothelial dysfunctions, especially those elicited by oxidized low-density lipoproteins (ox-LDL), play a critical role in the pathogenesis of AS. ox-LDL promotes vascular dysfunction by exerting cytotoxicity directly on endothelial cells and by enhancing the production of inflammatory mediators, including ROS, proinflammatory cytokines and arachidonic acid metabolites [4]. Ameliorating the oxidative stress state can be a key therapeutic strategy against AS.

The Zingiberaceae is a large diverse family comprising 1,200 species belonging to 49 genera. A prominent member of this family is the *Alpinia* genus. Plants of this genus are used in herbal medicine all over the world. *Alpinia zerumbet*, also known as *Alpinia speciosa* or *Alpinia nutans*[5], is an aromatic perennial ginger herb originating in the East Indies and naturalized in the subtropical and tropical regions of South America, Oceania and Asia [6]. In phytotherapy, *A. zerumbet* has been shown to possess pharmacological activities, including as an antioxidant and in the treatment of various conditions such as intestinal disorders, hypertension and inflammation [7,8].

In China, *A. zerumbet* is named Yanshanjiang, and has been used as a very valuable herbal medicine for hundreds of years. The herbalists used to separate each part of the *A. zerumbet* plant and apply them separately to different therapeutic uses. In particular as local *Miao* folk herbs in Guizhou province, the fruit was widely used against cardiovascular diseases. The essential oil from Fructus A. zerumbet (EOFAZ) has been shown to have major medicinal effects involving anti-inflammation, protection against cardiovascular diseases and antihypertension, as well as antioxidant activity [9,10]. Also, our previous research confirmed that the EOFAZ vasorelaxant effects depend on the endothelium-intact [11]. In another study we showed that compared to that of the fruit, the composition of the essential oil from the leaves is significantly different [12]. It is well known that ox-LDL-induced endothelial cell injury is a key pathological process in atherosclerosis. Considering all these aspects, especially the EOFAZ endothelium ameliorating function and the antiatherosclerosis effect, the present study was undertaken to evaluate the amelioration of the cellular redox status by EOFAZ for prevention of ox-LDL-induced endothelial cell injury.

## Methods

### Chemical and herbal materials

The essential oil was extracted from the fruit of *A. zerumbet*, which was collected in Zhenfeng county, Guizhou province, China, in October 2009. The fruit was identified by Professor Chen Zu-yun, and a voucher specimen (No.20091026) was deposited at the Research Division of Pharmacology, Guiyang Medical University. The isolation of the essential oil was carried out at the school of Pharmacy of the Guiyang Medical University, according to a method described elsewhere [13]. Briefly, chopped fruit was placed in a glass flask connected at one end to a glass vessel with water and at the other end to a water-cooled condenser. The water was heated to boiling point, and the steam percolated through the chopped fruit and collected in the condenser. After condensation, the watery phase with its solutes, termed the ‘hydrolate’, was separated from the oily phase; the essential oil when re-diluted in water is termed the ‘pseudo-hydrolate’. Sixty-two compounds were separated and 58 were identified from the essential oil. The composition of EOFAZ was determined by gas chromatography and mass spectrometry: β-phellandrene (16.388%), β-pinene (15.056%), 1,8-cineole (10.956%), camphene (10.120%), α-pinene (9.275%), linalool L (4.026%), camphor (3.657%), O-cymene (3.384%), β-myrcene (3.189%), borneol L (2.446%), caryophyllene oxide (1.778%) and terpinen-4-ol (1.756%); over 12 volatile compounds accounted for 82.03% of the total [12]. Pravastatin sodium standard substance (PRA) was purchased from the National Institute for the Control of Pharmaceutical and Biological Products of China, Beijing.

### Cell culture and treatments

Cell cultures were carried out essentially as described previously [14,15], with some modifications. Endothelial cells were isolated and pooled from human umbilical cords obtained less than 3 h after delivery (cords were obtained from three healthy puerperae donors, who gave informed consent, and the acquisition of tissues was carried out in line with the principles of the Declaration of Helsinki and all procedures with patients were approved by the ethical review board of Guiyang Medical University according to local and national guidelines). After rinsing, the veins were cannulated and incubated with 50 U/ml collagenase (Sigma-Aldrich, St. Louis, MO, USA) in serum-free M199 medium for 10 min. Primary cultures were seeded at a concentration of approximately 4×10^4^ cells/cm^2^ onto 75-cm^2^ flasks precoated with 0.01% (w/v) polygeline in phosphate-buffered saline (PBS). The culture medium was used M199 with Earle’s salts (Sigma-Aldrich), containing 20% (v/v) human serum (Shanghai Hengyuan, Shanghai, China), 5 U/ml penicillin G, 5 pg/ml streptomycin sulfate and 150 pg/ml endothelial cell growth supplement (Sigma-Aldrich). Cells reached confluency within approximately 4 d, and were cultured at 37°C in a humidified atmosphere with 5% CO_2_. The medium was changed a day after seeding and every 2 d thereafter.

For all experiments, cells were used at passages 3 to 6 and seeded at a concentration of 1×10^5^ cells/ml in 96-well plates, 24-well plates, or six-well plates. Treatments were carried out on a confluent monolayer of cells. EOFAZ was freshly prepared as a stock solution in DMSO and diluted with culture medium. DMSO was present at an equal concentration (0.02% final concentration) in all groups except for the PRA group. ox-LDL was used for no more than 2 wk from the date of production (Yiyuan Biotechnology, Guangzhou, China). Cells were preincubated for 30 min with EOFAZ or PRA before being exposed to 100 mg/L ox-LDL for 24 h except for the control group. After treatment, the cells cultured in the 96-well plates were used for the 3-(4,5-dimethylthiazol-2-yl)-2,5-diphenyltetrazolium bromide (MTT; Sigma-Aldrich) assay, those in the six-well plates were used for trypan blue (Beyotime, Haimen, China) exclusion staining (TBES), and those in the 24-well plates were assayed for the contents of malondialdehyde (MDA), glutathione (GSH) and LDH, and for the anti-oxidative enzymatic activity of superoxide dismutase (SOD), catalase (CAT) and glutathione peroxidase (GSH-Px). All experiments were performed in triplicate (four or five cultures were used for the experiments), however, the TBES assay was performed only once, but on four cultures.

### Cell viability measurements by the MTT assay and TBES

Cell viability was measured by the MTT reduction assay as previously described [16]. Cells from at least six wells were used for each group. Briefly, after a 24 h exposure to ox-LDL (100 mg/L), the supernatant was removed and the cells were gently washed with PBS followed by 80 μl of fresh culture medium and addition of 20 μl MTT (5 mg/ml). After 4 h of incubation, 200 μl of DMSO, the solubilization/stop solution, was added to dissolve the formazan crystals, and the absorbance was read by a microplate reader (Sunrise RC, Tecan, Switzerland) at a wavelength of 570 nm. Inhibition of cell damage (%) was calculated with the following formula:

(1) $$\text{Inhibition of cell damage}\;\left(\%\right)=\left({\text{OD}}_{\text{treated group}}-{\text{OD}}_{\text{ox}-\text{LDL group}}\right)/\left({\text{OD}}_{\text{control group}}-{\text{OD}}_{\text{ox}-\text{LDL group}}\right)\times 100$$

The TBES method is another classic cell viability method [17]. HUVECs in six-well plates were pretreated with EOFAZ or PRA for 30 min and then stimulated with 100 mg/L ox-LDL for 24 h. Then the cells were harvested and centrifuged to remove the medium. Cells were washed three times with PBS and resuspended to make a suspension of 1×10^6^ cells/ml. The suspension was mixed with 0.4% trypan blue dye for 5 min at 25°C, and the unstained (viable) and stained (nonviable) cells were counted on a hemacytometer within 5 min in five 40× microscope fields per well.

### LDH activity in the medium

To evaluate cell injury, the LDH released from the cytosol into the culture medium was measured as previously described [18]. After treatment with EOFAZ or PRA, followed by incubation with 100 mg/L ox-LDL for 24 h, the medium was collected from each well. Supernatants were obtained by centrifugation at 12,000×g at 4°C for 10 min. LDH release was determined using an LDH assay kit according to the manufacturer’s instructions (Nanjing Jiancheng Co., Nan Jing, China). In brief, 100 μl supernatant, 250 μl buffer and 50 μl coenzyme were mixed and incubated for 15 min at 37°C, followed by addition of 250 μl 2,4-dinitrophenylhydrazine and another 15 min incubation at 37°C in the dark. Finally, 2.5 ml NaOH (0.4 mol/L) were added to the reaction mixture. Three minutes later, 200 μl of each reaction mixture were transferred into a new 96-well plate. The absorbance was read at 440 nm with an ELISA reader.

### Cell lysate preparation

HUVECs were pretreated with EOFAZ or PRA for 30 min and then incubated with 100 mg/L ox-LDL for 24 h in 24-well plates. The cells were lysed with extraction buffer (20 mM Tris–HCl, pH 7.5, 150 mM NaCl, 1 mM EDTA, 1 mM EGTA, 1% Triton X-100, 2.5 mM sodium pyrophosphate, 1 mM glycerophosphate, 1 mM Na_3_VO_4_, 1 μg/ml leupeptin and 1 mM PMSF). Cell lysates from each well were collected and used for determination of MDA and GSH contents and anti-oxidative enzymatic activity. Protein concentrations of cell extracts were determined by the BCA assay (Santa Cruz Biotechnologies, Santa Cruz, CA, USA).

### Measurement of malondialdehyde (MDA) and glutathione (GSH) concentrations

The total levels of MDA, the lipid peroxidation product in the lysates, were measured by the thiobarbituric acid-reactive substance (TBARS) assay as described previously [19]. The lysates (100 μl) were mixed in glass test tubes with 3 ml of 1% phosphoric acid, 1 ml of 0.67% thiobarbituric acid and 0.04% butylated hydroxytoluene, and the mixtures were incubated in a boiling water bath for 60 min. Marbles were placed on the tops of the tubes during the incubation period to avoid excessive loss of reaction mixture. After cooling the tubes in ice, 1.5 ml of *n*-butanol were added and the reaction mixture was centrifuged at 1000×g for 10 min. The absorbance of the supernatant was read at 535 nm. The concentrations of TBARS were calculated using tetraethoxypropane as a reference standard. Results were expressed in nmol/mg protein.

Intracellular GSH was measured using the enzymatic recycling assay [20]. Aliquots (20 μl) were mixed with 100 mM TEA, 600 mM DTNB, and 210 mM NADPH in 6.3 mM EDTA-0.125 mM NaH_2_PO_4_, pH 7.5 buffer. The final volume was 200 μl and the reaction was initiated by adding 0.1 units GSH reductase. Absorbance was read at 405 nm in a 96-well plate format using an ELX800 microplate reader (Bio Tek, Winooski, VT, USA), and concentrations were determined using purified GSH as standard and expressed as nmoles of GSH per mg of protein (nmol/mg protein).

### Activity assay of anti-oxidative enzymes

SOD activity was measured by the xanthine/xanthine oxidase mediated ferricytochrome c reduction assay [21]. One unit of SOD activity was defined as the amount that reduced the absorbance at 550 nm by 50%. Fifty microliters of lysates were added to 2.9 ml of reaction buffer (0.5 μmol xanthine, 0.1 mM NaOH and 2 μmol cytochrome c in 50 mM K_2_HPO_4_-Na_2_HPO_4_/0.1 mM EDTA, pH 7.8). The reaction was initiated by adding 50 μl of xanthine oxidase solution (0.2 U/ml in 0.1 mM EDTA). The absorbance change was monitored for 3 min at 25°C. Activities were calculated using a concurrently run standard curve and expressed per mg of protein. The SOD activity results were expressed as U/mg protein.

The GSH-Px activity assay was conducted by quantifying the rate of oxidation of the reduced glutathione (GSH) to the oxidized glutathione by H_2_O_2_, which is catalyzed by GSH-Px. One unit of GSH-Px was defined as the amount that reduced the level of GSH by 1 μmol/L in 1 min per mg protein. GSH-Px activity was measured in a 1.0 ml cuvette containing 400 μl of 0.25 M potassium phosphate buffer (pH 7.0), 200 μl of sample, 100 μl of 10mM GSH, 100 μl of 2.5 mM NADPH and 100 μl of glutathione reductase (6 U/ml). Hydrogen peroxide (100 μl of 12 mM) was then added, and the change in absorbance was measured at 1min intervals for 5 min at 366 nm [22]. GSH-Px activity was expressed as U/mg protein compared to the standard.

CAT activity was detected by methods of our lab [23]. Briefly, 2.8 ml of 10 mmol/L H_2_O_2_ were added to a 5 ml cuvette that contained 0.2 ml of 1 mol/L Tris–HCl (pH 8.0) in an ice-water bath, and absorbance was measured immediately at 240 nm, which was OD_0_. Then 10 μl of lysate were added to the cuvette. The mixture was incubated at 25°C for 4 min, and then absorbance was measured, which was OD_4_. For the nonenzymatic reaction cuvette, the procedure was the same as for the enzymatic cuvette, but the cell lysate supernatant was substituted by distilled water, which was OD_n_. The results were expressed as catalytic activity following the equation: U/mg protein = (OD_0_ − OD_4_ − OD_n_) × CH_2_O_2_/C_protein_ × 4.

### Statistical analysis

Data were expressed as mean ± S.E.M (standard error of the mean) of at least three independent experiments. The differences in mean values among the experimental groups were measured using two-tailed analysis of variance (ANOVA) followed by Dunnett’s test. Differences were considered statistically significant at *p* < 0.05.

## Results

### EOFAZ attenuates the MTT reduction and blue staining ratio of HUVECs induced by ox-LDL

**Figure 1:**
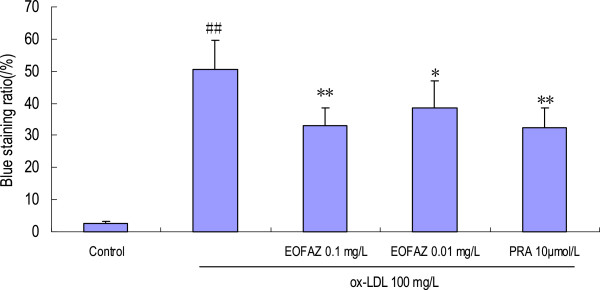
**Effects of EOFAZ on trypan blue exclusion staining in HUVECs' injury induced by ox-LDL.** HUVECs were pretreated for 30 min with EOFAZ or PRA, and then exposed to 100 mg/L ox-LDL for 24 h. A cell suspension was mixed with a 0.4% trypan blue dye for 5 min at 25°C, and the unstained (viable) and stained (nonviable) cells were counted in a hemacytometer. The values shown are mean ± S.E.M. of three experiments (four or five cultures per experiment). ^##^*p* < 0.01, vs. control group; *p < 0.05, **p < 0.01, vs. ox-LDL group.

The MTT assay utilizes the yellow MTT, which is metabolized by the mitochondrial succinate dehydrogenase to yield a purple formazan reaction product. Exposure of endothelial cells to 100 mg/L ox-LDL for 24 h significantly reduced cell viability (Table 1; 1.85 ± 0.11 in control vs. 0.98 ± 0.12 in 100 mg/L ox-LDL alone, *p* < 0.01). Preincubation with 0.1 mg/L EOFAZ restored viability significantly, but 0.01 mg/L EOFAZ had no effect (1.25Â±0.20 and 1.09Â±0.25 vs. 0.98Â±0.12, respectively, *p*<0.01 and *p*>0.05; Table 1). A similar protective effect was observed in the PRA treated group. The potential toxicity of EOFAZ to HUVECs was examined by addition of EOFAZ at 0.01, 0.1, 1 and 10 mg/L to the vehicle control medium. These concentrations had no effect on the survival of HUVECs, except for the 10 mg/L that led to a significant reduction in viability data were not shown. These results suggest that EOFAZ at concentrations of up to 1 mg/L is effective and safe to use with HUVECs. The trypan blue staining exclusion test is widely used to determine the number of viable cells present in a cell suspension. After incubation with 100 mg/L ox-LDL for 24 h, the number of stained cells significantly increased compared with the control group, and EOFAZ-pretreatment significantly decreased the cell blue staining ratio (Figure 1).

**Table 1:** Protective effects of EOFAZ on HUVECs’ injury induced by ox-LDL

**Groups**	**Dose (mg/L)**	**OD**_**570**_	**Inhibition of cell damage (%)**
Control	—	1.85±0.11	
ox-LDL	100	0.98±0.12^# #^	
ox-LDL+EOFAZ	100+0.1	1.25±0.20**	31.0
	100+0.01	1.09±0.25	12.6
ox-LDL+PRA	100+10 μmol/L	1.19±0.21*	24.1

^# #^*p* < 0.01, compared with the control group; *p < 0.05 and ** *p* < 0.01, compared with the ox-LDL group.

### EOFAZ inhibits LDH release from cells treated with ox-LDL

**Figure 2:**
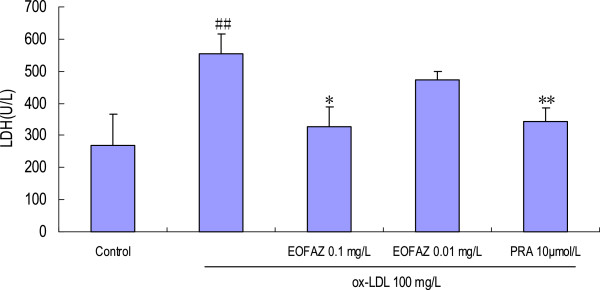
**Effects of EOFAZ on LDH activity in HUVECs' injury induced by ox-LDL.** HUVECs were treated as described in Figure 1, and then the culture medium was collected to examine LDH activity. The values shown are mean ± S.E.M. of three experiments (four or five cultures per experiment). ^##^*p* < 0.01, vs. control group; *p < 0.05, **p < 0.01, vs. ox-LDL group.

A 24 h exposure to 100 mg/L ox-LDL resulted in a marked and significant facilitation of LDH release from the cells, compared with the vehicle control group (555.15±59.67 U/L vs. 269.12 ± 97.80 U/L, *p* < 0.01). In contrast, the LDH activity in the supernatant was significantly decreased in the samples pretreated with 0.1 mg/L EOFAZ compared with the samples treated with ox-LDL alone (328.68 ± 61.29 U/L vs. 555.15±59.67 U/L, *p* < 0.01; Figure 2).

### EOFAZ reduces the MDA contents and increases the GSH contents in cells treated with ox-LDL

**Figure 3:**
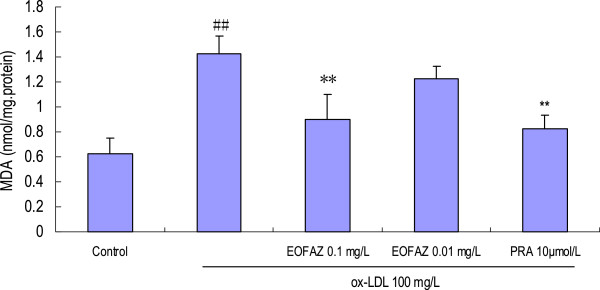
**Effects of EOFAZ on MDA contents in HUVECs' injury induced by ox-LDL.** HUVECs were treated as described in Figure 1, followed by cell lysate preparation. MDA contents was measured with the thiobarbituric acid-reactive substance (TBARS) assay. The values shown are mean ± S.E.M. of three experiments (four or five cultures per experiment). ^##^*p* < 0.01, vs. control group; **p < 0.01, vs. ox-LDL group.

**Figure 4:**
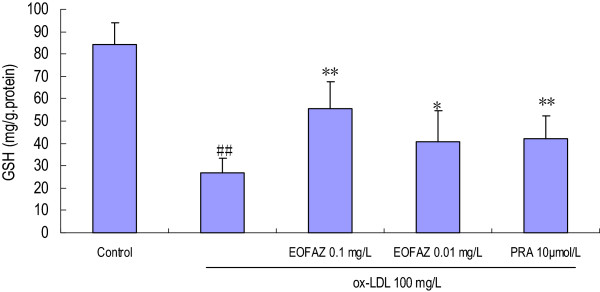
**Effects of EOFAZ on GSH contents in HUVECs' injury induced by ox-LDL.** HUVECs treatment and cell lysate preparation were performed as described in Figure 3. The enzymatic recycling assay was used to detect the GSH contents. The values shown are mean ± S.E.M. of three experiments (four or five cultures per experiment). ^##^*p* < 0.01, vs. control group; *p < 0.05, **p < 0.01, vs. ox-LDL group.

In the present study, incubation of HUVECs with 100 mg/L ox-LDL for 24 h resulted in a significant increase in the MDA contents, which was significantly attenuated by EOFAZ or PRA pretreatment (Figure 3). However, the exposure of HUVECs to ox-LDL 100 mg/L for 24 h resulted in a significant decrease in GSH contents, which was ameliorated by preincubation with EOFAZ or PRA (Figure 4).

### EOFAZ upregulates the activities of antioxidant enzymes

**Figure 5:**
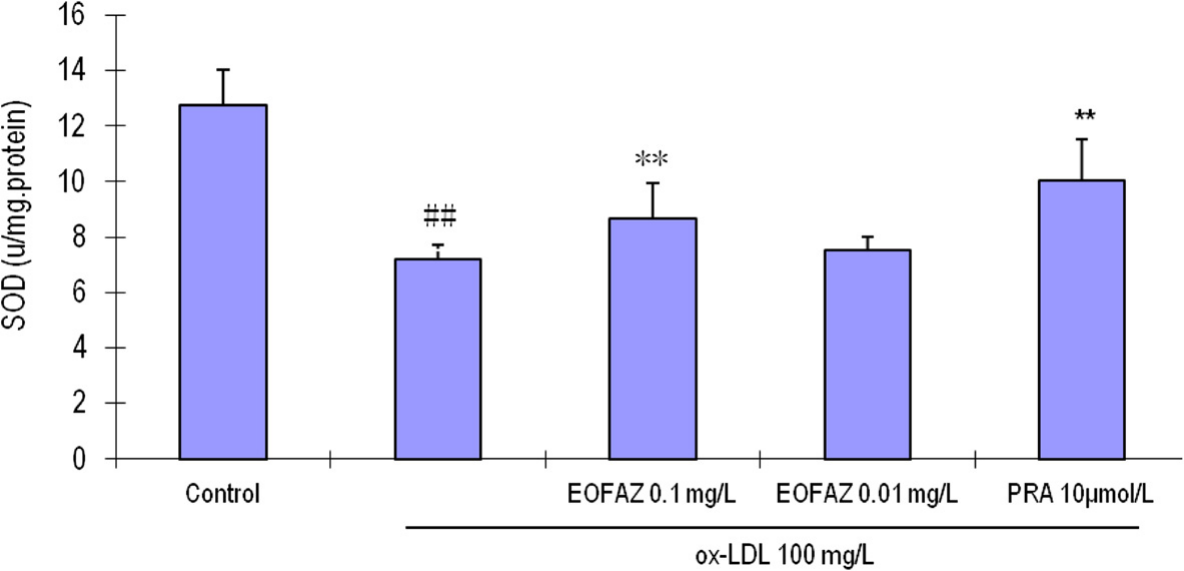
**Effects of EOFAZ on SOD activity in HUVECs exposed to ox-LDL.** HUVECs treatment and cell lysate preparation were performed as described in Figure 3. SOD activity was measured by the xanthine/xanthime oxidase mediated ferricytochrome c reduction assay. The values shown are mean ± S.E.M. of three experiments (four or five cultures per experiment). ^##^*p* < 0.01, vs. control group; **p < 0.01, vs. ox-LDL group.

**Figure 6:**
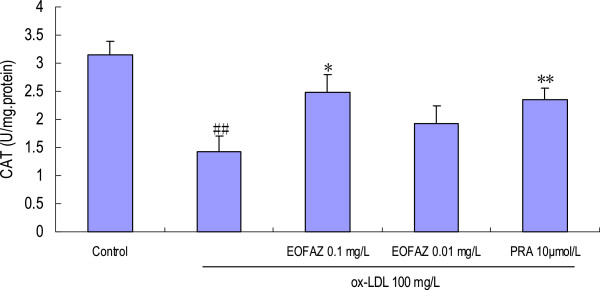
**Effects of EOFAZ on CAT activity in HUVECs exposed to ox-LDL.** HUVECs treatment and cell lysate preparation were performed as described in Figure 3. CAT activity was assayed as described in the Methods section. The values shown are mean ± S.E.M. of three experiments (four or five cultures per experiment). ^##^*p* < 0.01, vs. control group; **p < 0.01, vs. ox-LDL group.

**Figure 7:**
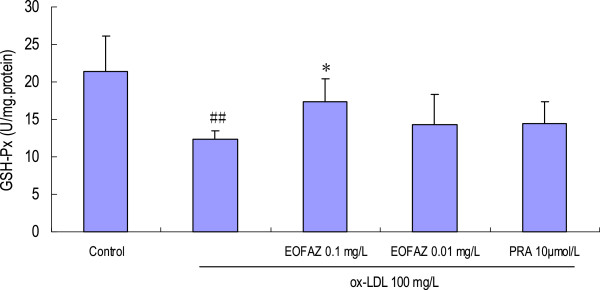
**Effects of EOFAZ on GSH-Px activity in HUVECs exposed to ox-LDL.** HUVECs treatment and cell lysate preparation were performed as described in Figure 3. GSH-Px activity was determined by quantifying the rate of oxidation of the reduced glutathione (GSH) to the oxidized glutathione (GSSG) by H_2_O_2_, which is catalyzed by GSH-Px. The values shown are mean ± S.E.M. of three experiments (four or five cultures per experiment). ^##^*p* < 0.01, vs. control group; *p < 0.05, vs. ox-LDL group.

The main scavengers responsible for inactivation and termination of free oxygen radicals are SOD, CAT, and the glutathione system. Our results indicated that the activities of the anti-oxidative enzymes were significantly decreased after HUVECs were exposed to 100 mg/L ox-LDL, and that EOFAZ and PRA improved these activities (Figures 5, 6 and 7). Preincubation with 0.1 mg/L EOFAZ significantly ameliorated the activities of SOD, CAT and GSH-Px in the cell lysates. Preincubation with 0.01 mg/L EOFAZ enhanced the activities of these enzymes, however, these results were not statistically significant. PRA (10 μmol/L) alleviated the decreased activities of SOD and CAT (**p < 0.01, compared with the ox-LDL treatment), but did not improve the GSH-Px activity.

## Discussion

To the best of our knowledge, the present study demonstrates for the first time that EOFAZ (the composition was significantly different from previous reports and from the essential oil extract from the fruit of A. *zerumbet*) protects against endothelial cell injury induced by ox-LDL via ameliorating oxidative stress, which may be the main underlying mechanism.

Human umbilical vein endothelial cells (HUVECs) have played a major role as a model system for studying the regulation of endothelial cell function and the role of the endothelium in the response of the blood vessel wall to stretch, shear forces, and the development of atherosclerotic plaques and angiogenesis [24]. It is well known that the atherosclerotic lesion is characterized by an accumulation of lipids carried by lipoproteins, such as low-density lipoprotein (LDL). LDL becomes susceptible to (non)enzymatic oxidative modifications when retained in the artery wall [25]. These modifications make LDL a potent effector of cellular functions. Multiple lines of evidence suggest that oxidative stress, characterized by an elevated generation of ROS, is involved in the pathogenesis of AS, which implies that oxidized-LDL may promote the development of AS through oxidative stress, and is one of the most important risk factors for AS and cardiovascular morbidity [26]. Endothelial dysfunction elicited by ox-LDL has been demonstrated as the key step in the initiation of AS. It is widely accepted that ox-LDL-induced endothelial dysfunction is associated with an alteration of the cell redox status, and ameliorating the redox status has been a key therapeutic strategy against AS in the clinic [27].

The cell injury was evaluated by the MTT assay, TBES, and LDH release, which are widely accepted methods for cell injury evaluation. The MTT assay is a quantitative colorimetric method to determine cell proliferation or injury. It utilizes the yellow MTT, which is metabolized by the mitochondrial succinate dehydrogenase to yield a purple formazan reaction product. The TBES method is widely used to determine the number of viable cells and is based on the principle that live cells possess intact cell membranes that exclude certain dyes, such as trypan blue, eosin and propidium, whereas dead cells do not. Lactate dehydrogenase (LDH) is a stable enzyme in the cytosol, present in all cell types, that is rapidly released into the medium upon damage of the plasma membrane; hence it is a biomarker for cell membrane damage. The cell injury induced by incubation with ox-LDL for 24 h was confirmed by the MTT assay (OD_570_ decrease), trypan blue staining ratio increase, and the LDH activity increase. Pretreatment with EOFAZ or PRA ameliorated the cell injury.

Membranes of bioplasm are very rich in polyunsaturated fatty acids, which are especially sensitive to free radical-induced lipid peroxidation. In oxidative stress, superoxide anion and hydrogen peroxide are formed and cannot be readily scavenged because of the low activities of CAT, SOD and GSH-Px in the endothelial cell [28]. Augmentation of endogenous antioxidants (SOD, CAT and GSH-Px) has been recognized as an important pharmacological property present in natural as well as many synthetic compounds [29]. This constitutes a major mechanism of protection against oxidative stress [30,31]. The most abundant ROS generated in living systems is the superoxide radical, which is acted upon by SOD to produce hydrogen peroxide, which in turn is broken down by catalase and/or GSH-Px into water and oxygen. Thus, increase in both SOD and catalase along with GSH-Px activity is considered to be more beneficial in the event of oxidative stress.

Exposure to 100 mg/L ox-LDL for 24 h, increased the MDA contents, decreased the GSH contents, and inhibited the enzymes’ antioxidant activity, indicating that the redox status was exacerbated. In the present study, a 30 min preincubation with EOFAZ significantly elevated the activities of SOD, CAT and GSH-Px, whereas PRA only enhanced the activity of SOD and CAT. Obviously, EOFAZ scavenged hydrogen peroxide and superoxide anion, further decreasing the formation of hydroxyl radicals and attenuating lipid peroxidative damage after the HUVECs were exposed to ox-LDL. However, it is not clear whether EOFAZ induces the expression of the endogenous antioxidant enzymes or whether it has direct protective effects of endogenous antioxidants. PRA, a hydroxymethylglutaryl-CoA reductase inhibitor, is a member of the statins drug class and was used here as a positive control. PRA, which is used for lowering cholesterol and preventing cardiovascular disease, and is widely used to prevent AS, has anti-oxidative effects [32]. The present results confirmed previous research.

Oxidative metabolites are involved in the functional inactivation of endothelial cells by increasing permeability and as potent inducers of endothelial cell death. The level of MDA reflects the extent of cell damage by oxidative stress [33]. GSH is an intracellular reductant that plays major roles in catalysis, metabolism and transport, and protects cells against free radicals, peroxides, and other toxic compounds, as it is a critical factor involved in the glutathione system to scavenge hydrogen peroxide and organic hydroperoxides [34]. It is well known that a deficiency in GSH within living organisms can lead to tissue disorders and injury. The present study demonstrated that EOFAZ alleviated the increase in MDA and the decrease in GSH contents caused by the ox-LDL-induced endothelial cell damage, suggesting it has the potential to protect the membranes of HUVECs from lipid peroxidative damage.

Endothelial dysfunction is an early feature of both atherosclerosis and vascular diseases, which may lead to an improvement of prognosis in patients with cardiovascular risk factors preventing the development of atherosclerosis and consequently promoting a reduction in cardiovascular events [35]. Chronic inflammation and oxidative stress play crucial roles in endothelial dysfunction and atherosclerosis [36]. Controlling a variety of risk factors causing inflammation and oxidative stress with combination therapy may simultaneously address multiple mechanisms underlying the pathogenesis of atherosclerosis. EOFAZ has been shown to be an anti-inflammatory agent in previous research [37] and the current results confirmed it is also an antioxidant, which may provide a novel therapy for atherosclerosis in the clinic.

In summary, the present research indicates that increase in HUVECs’ lipid peroxidation and depletion of endogenous antioxidants support the occurrence of oxidative stress after exposure to ox-LDL. Furthermore, it was accompanied by cell viability decrease, which was confirmed by the MTT assay, trypan blue exclusion, and LDH release. EOFAZ protected the cells against oxidative stress, as evidenced by inhibition of the increased MDA contents and decreased GSH contents, alleviation of CAT, SOD and GSH-Px activities, and finally, enhancement of cell viability. The mechanism of this protection can be attributed to the augmented endogenous antioxidant reserve in HUVECs and/or a direct antioxidant effect. This study shows that EOFAZ from natural products may be an excellent protecting agent against endothelial cell injury in the clinic; the mechanism will be explored in detail and a clinical evaluation conducted in future research.

## Conclusions

The present findings provide the first experimental evidence that EOFAZ protects endothelial cells against ox-LDL-induced injury, and indicate that this protection involves amelioration of the redox status.

### Ethics approval

This study was approved by the ethical review board of Guiyang Medical University according to local and national guidelines. Care of the patients in this study was in accordance with the Declaration of Helsinki and all relevant laws.

## Abbreviations

AS: Atherosclerosis; CAT: Catalase; DMSO: Dimethyl sulfoxide; EOFAZ: Essential oil from Fructus Alpiniae Zerumbet; FAZ: Fructus Alpiniae Zerumbet; GSH: Reduced glutathione; GSH-Px: Glutathione peroxidase; HUVECs: Human umbilical vein endothelial cells; LDH: Lactate dehydrogenase; MDA: Malondialdehyde; MTT: 3-(4,5-dimethylthiazol-2-yl)-2,5-diphenyltetrazolium bromide; OD: Optical density; ox-LDL: Oxidized low-density lipoprotein; PBS: Phosphate-buffered saline; ROS: Reactive oxygen species; PRA: Pravastatin; SOD: Superoxide dismutase; TBES: Trypan blue exclusion staining.

## Competing interests

The authors declare that they have no competing interests.

## Authors’ contributions

XS: Supervised the work, provided the grant, designed and performed the study, evaluated the data, prepared and corrected the manuscript, and coordinated the study. LT: Prepared the essential oil from Fructus Alpiniae Zerumbet and performed the biochemical assays. WL: Performed the study and analyzed the data. YZ, HL and YX: Performed the biochemical assays. All authors read and approved the final manuscript.

## Pre-publication history

The pre-publication history for this paper can be accessed here:

http://www.biomedcentral.com/1472-6882/12/174/prepub

## References

[B1] Stocker R Keaney FJ Role of oxidative modifications in atherosclerosis Physiol Rev 2004 84 4 1381 1478 10.1152/physrev.00047.2003 15383655

[B2] Fotis L Agrogiannis G Vlachos IS Pantopoulou A Margoni A Kostaki M Verikokos C Tzivras D Mikhailidis DP Perrea D Intercellular adhesion melecule ( ICAM )-1 and vascular cell adhesion molecule ( VCAM )-1 at the early stages of atherosclerosis in a rat model In Vivo 2012 26 2 243 250 22351665

[B3] Lum H Roebuck KA Oxidant stress and endothelial cell dysfunction Am J Physiol Cell Physiol 2001 280 4 C719 C741 11245588 10.1152/ajpcell.2001.280.4.C719

[B4] Mitra S Deshmukh A Sachdeva R Lu J Mehta JL Oxidized low-density lipoprotein and atherosclerosis implications in antioxidant therapy Am J Med Sci 2011 342 2 135 142 10.1097/MAJ.0b013e318224a147 21747278

[B5] Mendonca VL Oliveira CL Craveiro AA Rao VS Fonteles MC Pharmacological and toxicological evaluation of Alpinia specosa Mem Inst Oswaldo Cruz 1991 86 Suppl 2 93 97 1842022 10.1590/s0074-02761991000600023

[B6] Murakami S Li W Matsuura M Composition and seasonal variation of essential oil in Alpinia zerumbet from Okinawa Island J Nat Med 2009 63 2 204 208 10.1007/s11418-008-0306-4 19067113

[B7] Yob NJ Jofrry SM Affandi MM The LK Sallen MZ Zakaria ZA Zingiber zerumbet (L.) Smith: a review of its ethnomedicinal, chemical, and pharmacological uses Evid Based Complement Alternat Med 2011 2011 543216 21584247 10.1155/2011/543216 PMC3092606

[B8] Chompoo J Upadhyay A Fukuta M Tawata S Effect of Alpinia zerumbet components on antioxidant and skin diseases-related enzymes BMC Complement Altern Med 2012 12 1 106 10.1186/1472-6882-12-106 22827920 PMC3419607

[B9] Chuang CM Wang HE Peng CC Chen KC Peng RY Hypolipidemic effects of different angiocarp parts of Alpinia zerumbet Pharm Biol 2011 49 12 1257 1264 10.3109/13880209.2011.589856 21846199

[B10] Lin LY Peng CC Liang YJ Yeh WT Wang HE Yu TH Peng RY Alpinia zerumbet potentially elevates high-density lipoprotein cholesterol level in hamsters J Agric Food Chem 2008 56 12 4435 4443 10.1021/jf800195d 18522401

[B11] Tao L Xiao TT Hu HS Tu L Liu XD Effects of volatile oils from fructus Alpiniae zerumber on the contraction of rabbit thoracic aorta in vitro Chin Hosp Pharm J 2010 30 23 1966 1969

[B12] Shen XC Hu HS Xiao HT GC-MS analysis of chemical constituents of the essential oil from different parts of Alpinia zerumbet (Pers.) Burtt et Smith Chin J Pharm Anal 2010 30 8 1399 1403

[B13] de Araújoa FYR de Oliveiraa GV Gomesa PXL Soaresa MA Silvaa MIG Carvalhob AF de Moraesa MO de Moraesa MEA Vasconcelosa SMM Vianaa GSB de Sousaa FCF Macêdoa DS Inhibition of ketamine-induced hyperlocomotion in mice by the essential oil of Alpinia zerumbet: possible involvement of an antioxidant effect J Pharm Pharmacol 2011 63 8 1103 1110 21718294 10.1111/j.2042-7158.2011.01312.x

[B14] Zhang YW Morita I Nishida M Murota SI Involvement of tyrosine kinase in the hypoxia/reoxygenation-induced gap junctional intercellular communication abnormality in cultured human umbilical vein endothelial cells J Cell Physiol 1999 180 3 305 313 10.1002/(SICI)1097-4652(199909)180:3<305::AID-JCP1>3.0.CO;2-Z 10430170

[B15] Ou HC Lee WJ Lee IT Chiu TH Tsai KL Lin CY Sheu WHH Ginkgo biloba extract attenuates ox-LDL-induced oxidative functional damages in endothelial cells J Appl Physiol 2009 106 5 1674 1685 10.1152/japplphysiol.91415.2008 19228986

[B16] Lee SM Yoon MY Park HR Protective effects of Paeonia lactiflora pall on hydrogen peroxide-induced apoptosis in PC12 cells Biosci Biotechnol Biochem 2008 72 5 1272 1277 10.1271/bbb.70756 18460804

[B17] Alabsi AM Bakar SAA Ali R Omar AR Bejo MH Ideris A Ali AM Effects of Newcastle disease virus strains AF2240 and V4-UPM on cytolysis and apoptosis of leukemia cell lines Int J Mol Sci 2011 12 12 8645 8660 10.3390/ijms12128645 22272097 PMC3257094

[B18] Liu LN Mei QB Liu L Zhang F Liu ZG Wang ZP Wang RT Protective effects of Rheum tanguticum polysaccharide against hydrogen peroxide-induced intestinal epithelial cell injury World J Gastroenterol 2005 11 10 1503 1507 15770727 10.3748/wjg.v11.i10.1503 PMC4305693

[B19] Wang B Peng L Zhu L Ren P Protective effect of tota flavonoids from Spirodela polyrrhiza (L.) Schleid on human umbilical vein endothelial cell damage induced by hydrogen peroxide Colloids Surf B Biointerfaces 2007 60 1 36 40 10.1016/j.colsurfb.2007.05.020 17628450

[B20] Nakamura YK Dubick MA Omaye ST γ-Glutamylcysteine inhibits oxidative stress in human endothelial cells Life Sci 2012 90 3–4 116 121 22075492 10.1016/j.lfs.2011.10.016

[B21] Yang S Zhu H Li Y Lin H Gabrielson G Trush MA Diehl AM Mitochondrial adaptations to obesity-related oxidant stress Arch Biochem Biophys 2000 378 2 259 268 10.1006/abbi.2000.1829 10860543

[B22] Diehl AM Chacon MA Wagner P The effect of chronic ethanol feeding on ornithine decarboxylase activity and liver regeneration Hepatology 1988 8 2 237 242 10.1002/hep.1840080208 3356404

[B23] Shen XC Qian ZY Effects of crocetin on antioxidant enzymatic activities in cardiac hypertrophy induced by norepinephrine in rats Pharmazie 2006 61 4 348 352 16649553

[B24] Park HJ Zhang Y Georgescu SP Johnson KL Kong D Galper JB Human umbilical vein endothelial cells and human dermal microvascular endothelial cells offer new insights into the relationship between lipid metabolism and angiogenesis Stem Cell Rev 2006 2 2 93 102 10.1007/s12015-006-0015-x 17237547

[B25] Cappello C Saugel B Huth BS Zwergal HA Krautkaramer M Furman C Rouis M Wieser B Schneider HW Neumeier D Brand K Ozonized low density lipoprotein (ozLDL) inhibits NF-κB and IRAK-1-associated signaling Artherioscler Thromb Vasc Biol 2007 27 1 226 232 10.1161/01.ATV.0000250615.27795.85 17053167

[B26] Chowdhury SKR Sangle GV Xie XP Stelmack GL Halayko AJ Shen GX Effects of extensively oxidized low-density lipoprotein on mitochondrial function and reactive oxygen species in pocine aortic endothelial cells Am J Physiol Endocrinol Metab 2010 298 1 E89 E98 10.1152/ajpendo.00433.2009 19843872

[B27] Sharma A Bernatchez P de Haan JB Targeting endothelial dysfunction in vascular complications associated with diabetes Int J Vasc Med 2012 10.1155/2012/750126 PMC3195347 22013533

[B28] Drǒge W Free radicals in the physiological control of cell function Physiol Rev 2002 82 1 47 95 11773609 10.1152/physrev.00018.2001

[B29] Gauthman K Maulik M Kumari R Manchanda SC Dinda AK Maulik SK Effect of chronic treatment with bark of Terminalia arjuna: a study on the isolated ischemic-reperfused rat heart J Ethnopharmacol 2001 75 2–3 197 201 11297851 10.1016/s0378-8741(01)00183-0

[B30] Bhattacharya SK Bhattacharya A Sairam K Ghosal S Effect of bioactive tannoid principles of Emblica officinalis on ischemia-reperfusion-induced oxidative stress in rat heart Phytomedicine 2002 9 2 171 174 10.1078/0944-7113-00090 11995952

[B31] Rajak S Banerjee SK Sood S Dinda AK Gupta YK Gupta SK Maulik SK Emblica officinalis causes myocardial adaptation and protects against oxidative stress in ischemic-reperfusion injury Phytother Res 2004 18 1 54 60 10.1002/ptr.1367 14750202

[B32] Thallinger C Urbauer E Lackner E Graselli U Kostner K Wolzt M Joukhadar C The ability of statins to protect low density lipoprotein from oxidation in hypercholesterolemic patients Int J Clin Pharmacol Ther 2005 43 12 551 557 16372516 10.5414/cpp43551

[B33] Mei X Xu D Xu S Zheng Y Xu S Novel role of Zn (II)-curcumin in enhancing cell proliferation and adjusting proinflammatory cytokine-mediated oxidative damage of ethanol-induced acute gastric ulcers Chem Biol Interact 2012 197 1 31 39 10.1016/j.cbi.2012.03.006 22465177

[B34] Cacciatore I Cornacchia CC Pinnen F Mollica A Stefano AD Prodrug approach for increasing cellular glutathione levels Molecules 2010 15 3 1242 1264 10.3390/molecules15031242 20335977 PMC6257297

[B35] Grassi D Desideri G Ferri C Cardiovascular risk and endothelial dysfunction: the preferential route for atherosclerosis Curr Pharm Biotechnol 2011 12 9 1343 1353 10.2174/138920111798281018 21235459

[B36] Koh KK Oh PC Quon MJ Does reversal of oxidative stress and inflammation provide vascular protection? Cardiovasc Res 2009 81 4 649 659 19098298 10.1093/cvr/cvn354

[B37] Tao L Shen XC Peng J Bo S Experimental study on the anti-inflammatory and analgesic effects in vivo of essential oil from frucus Alpiniae zerumbet in mice Chin Hosp Pharm J 2010 30 9 722 724

